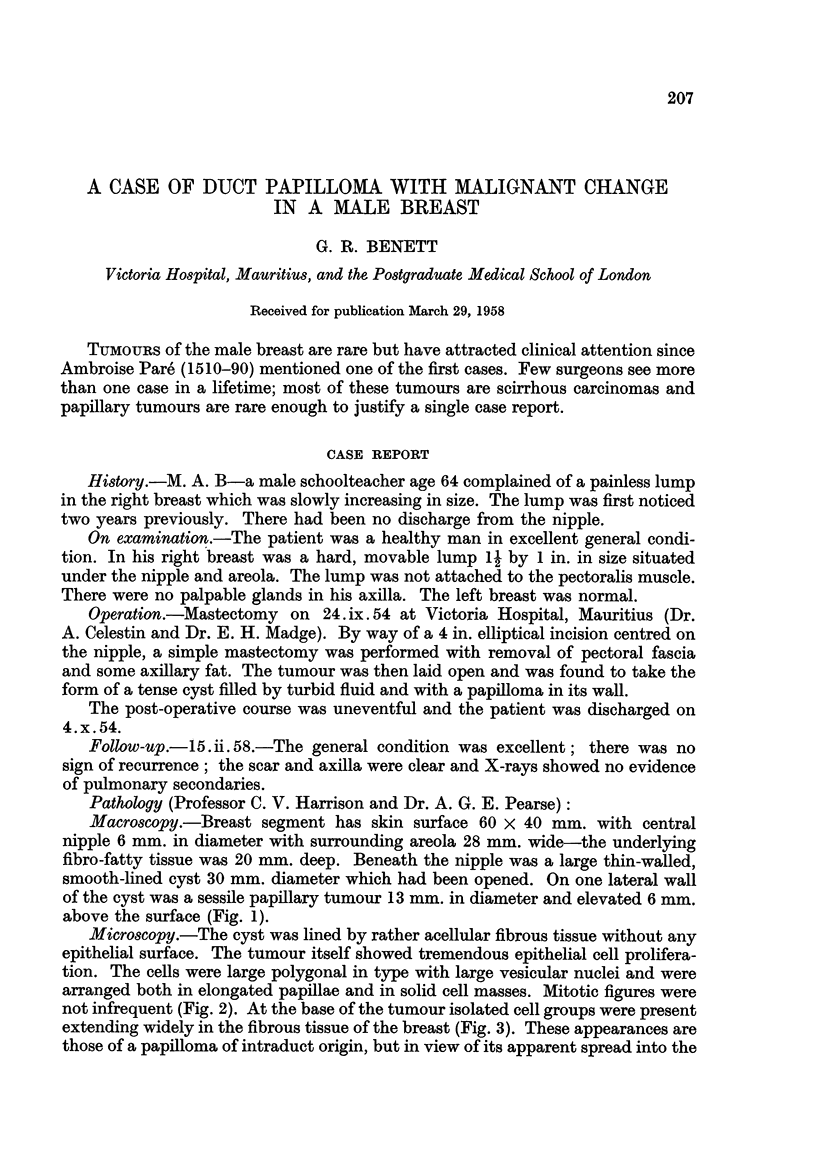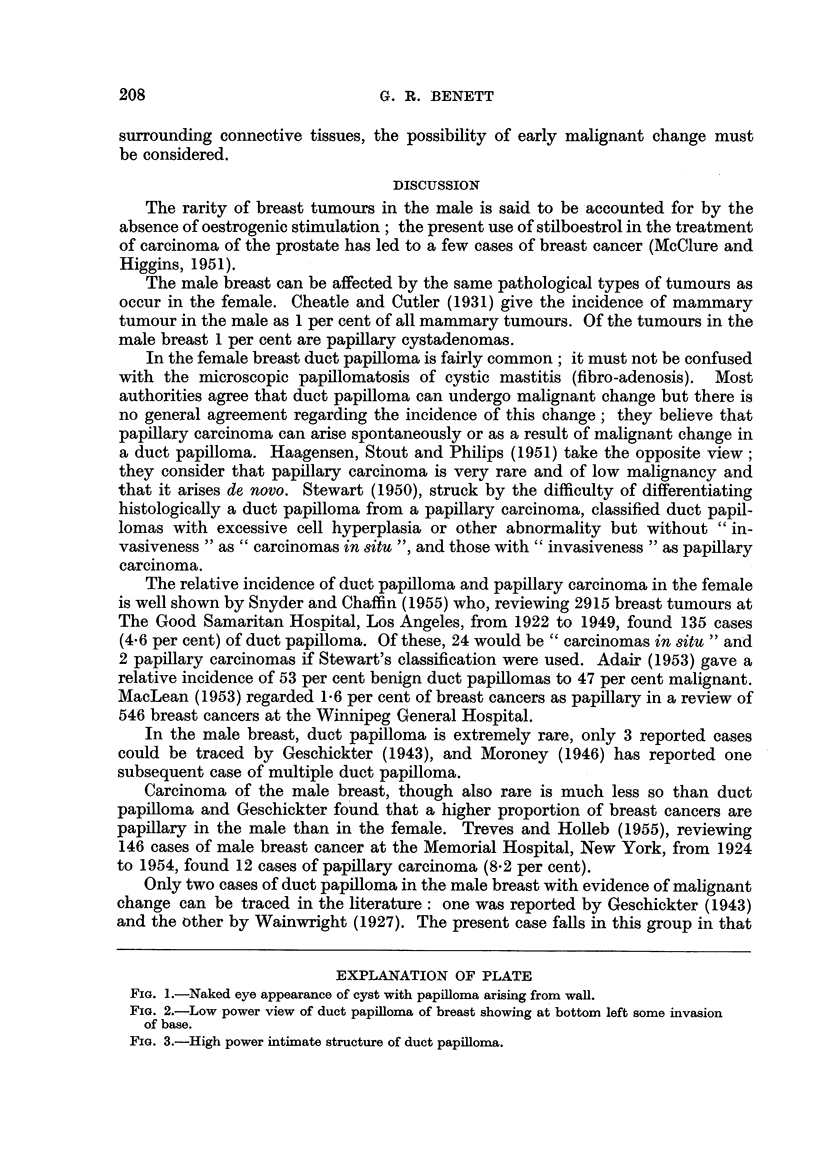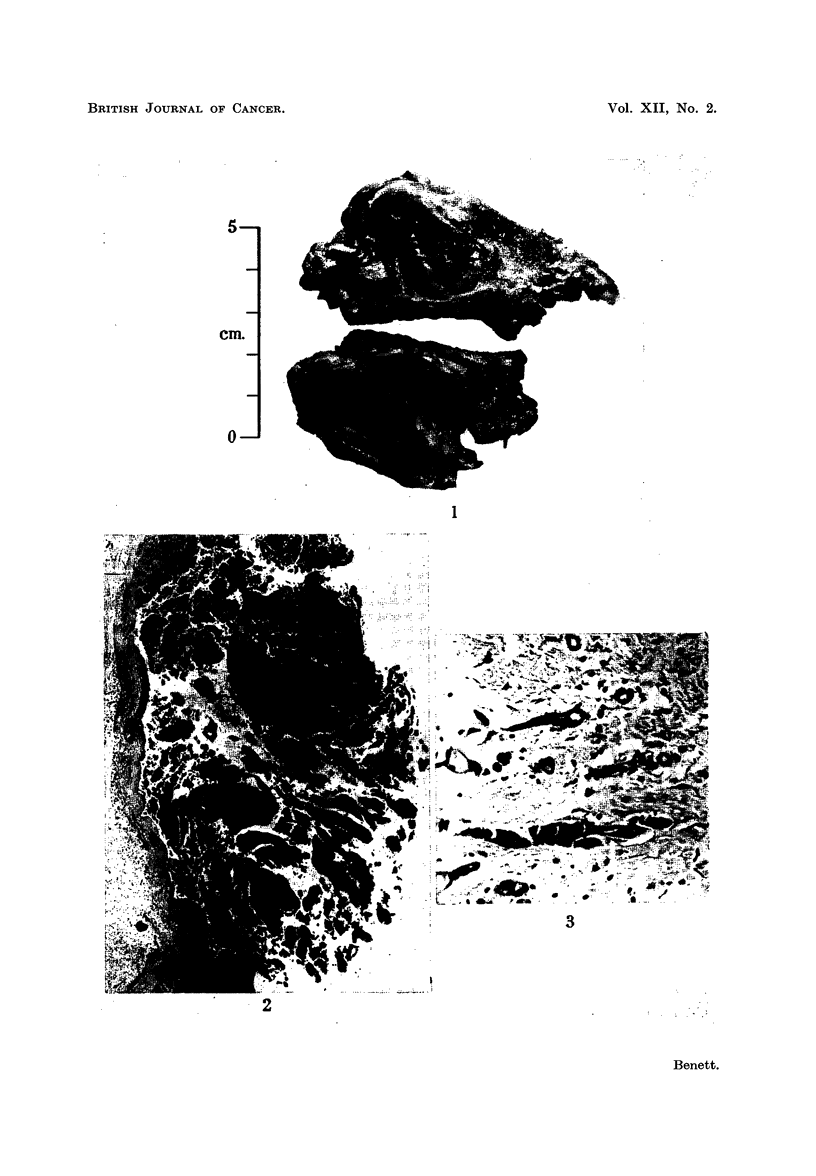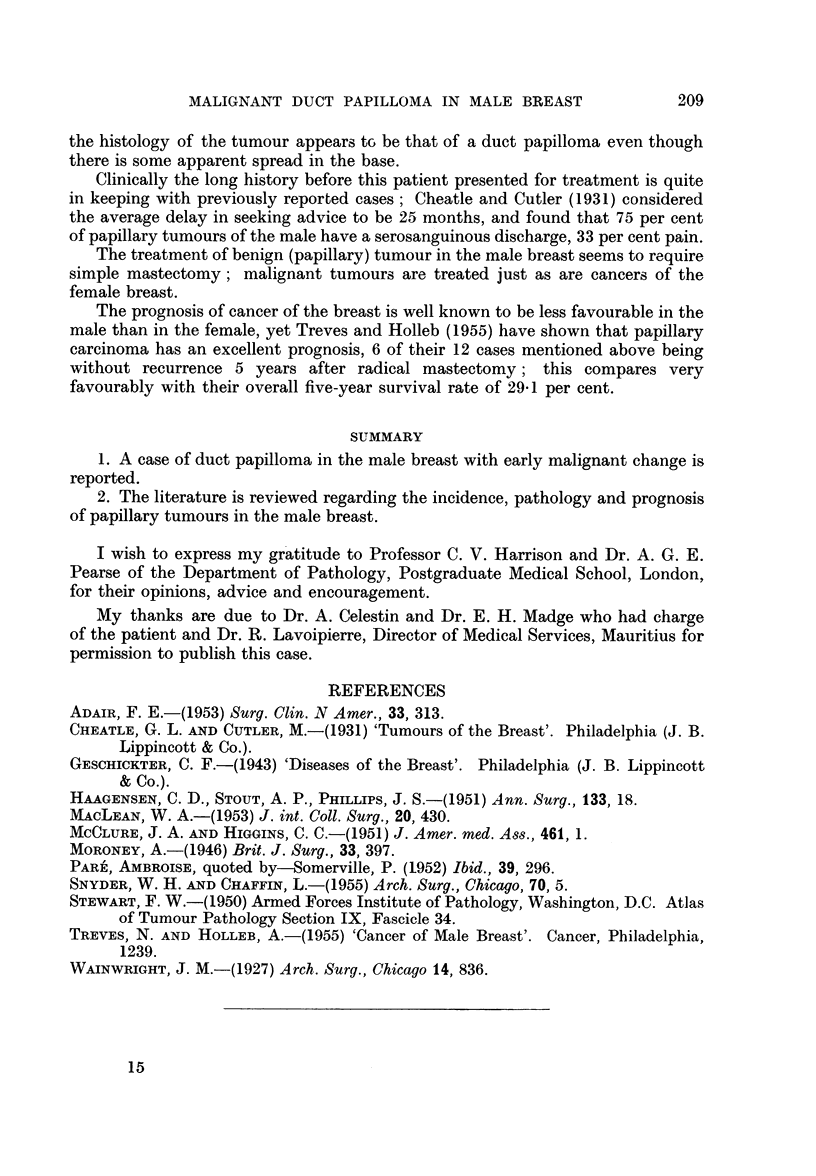# A Case of Duct Papilloma with Malignant Change in a Male Breast

**DOI:** 10.1038/bjc.1958.24

**Published:** 1958-06

**Authors:** G. R. Benett

## Abstract

**Images:**


### A CASE OF DUCT PAPILLOMA WITH MALIGNANT CHANGE IN A MALE BREAST

G. R. BENETT

*Victoria Hospital, Mauritius, and the Postgraduate Medical School of London*

Received for publication March 29, 1958

TUMOURS of the male breast are rare but have attracted clinical attention since Ambroise Paré (1510–90) mentioned one of the first cases. Few surgeons see more than one case in a lifetime; most of these tumours are scirrhous carcinomas and papillary tumours are rare enough to justify a single case report.

### CASE REPORT

*History.*—M. A. B—a male schoolteacher age 64 complained of a painless lump in the right breast which was slowly increasing in size. The lump was first noticed two years previously. There had been no discharge from the nipple.

*On examination.*—The patient was a healthy man in excellent general condition. In his right breast was a hard, movable lump $1\frac{1}{2}$ by 1 in. in size situated under the nipple and areola. The lump was not attached to the pectoralis muscle. There were no palpable glands in his axilla. The left breast was normal.

*Operation.*—Mastectomy on 24.ix.54 at Victoria Hospital, Mauritius (Dr. A. Celestin and Dr. E. H. Madge). By way of a 4 in. elliptical incision centred on the nipple, a simple mastectomy was performed with removal of pectoral fascia and some axillary fat. The tumour was then laid open and was found to take the form of a tense cyst filled by turbid fluid and with a papilloma in its wall.

The post-operative course was uneventful and the patient was discharged on 4.x.54.

*Follow-up.*—15.ii.58.—The general condition was excellent; there was no sign of recurrence; the scar and axilla were clear and X-rays showed no evidence of pulmonary secondaries.

*Pathology* (Professor C. V. Harrison and Dr. A. G. E. Pearse):

*Macroscopy.*—Breast segment has skin surface 60 × 40 mm. with central nipple 6 mm. in diameter with surrounding areola 28 mm. wide—the underlying fibro-fatty tissue was 20 mm. deep. Beneath the nipple was a large thin-walled, smooth-lined cyst 30 mm. diameter which had been opened. On one lateral wall of the cyst was a sessile papillary tumour 13 mm. in diameter and elevated 6 mm. above the surface (Fig. 1).

*Microscopy.*—The cyst was lined by rather acellular fibrous tissue without any epithelial surface. The tumour itself showed tremendous epithelial cell proliferation. The cells were large polygonal in type with large vesicular nuclei and were arranged both in elongated papillae and in solid cell masses. Mitotic figures were not infrequent (Fig. 2). At the base of the tumour isolated cell groups were present extending widely in the fibrous tissue of the breast (Fig. 3). These appearances are those of a papilloma of intraduct origin, but in view of its apparent spread into the surrounding connective tissues, the possibility of early malignant change must be considered.

### DISCUSSION

The rarity of breast tumours in the male is said to be accounted for by the absence of oestrogenic stimulation ; the present use of stilboestrol in the treatment of carcinoma of the prostate has led to a few cases of breast cancer (McClure and Higgins, 1951).

The male breast can be affected by the same pathological types of tumours as occur in the female. Cheatle and Cutler (1931) give the incidence of mammary tumour in the male as 1 per cent of all mammary tumours. Of the tumours in the male breast 1 per cent are papillary cystadenomas.

In the female breast duct papilloma is fairly common ; it must not be confused with the microscopic papillomatosis of cystic mastitis (fibro-adenosis). Most authorities agree that duct papilloma can undergo malignant change but there is no general agreement regarding the incidence of this change ; they believe that papillary carcinoma can arise spontaneously or as a result of malignant change in a duct papilloma. Haagensen, Stout and Philips (1951) take the opposite view ; they consider that papillary carcinoma is very rare and of low malignancy and that it arises *de novo*. Stewart (1950), struck by the difficulty of differentiating histologically a duct papilloma from a papillary carcinoma, classified duct papillomas with excessive cell hyperplasia or other abnormality but without " invasiveness " as " carcinomas *in situ* ", and those with " invasiveness " as papillary carcinoma.

The relative incidence of duct papilloma and papillary carcinoma in the female is well shown by Snyder and Chaffin (1955) who, reviewing 2915 breast tumours at The Good Samaritan Hospital, Los Angeles, from 1922 to 1949, found 135 cases (4·6 per cent) of duct papilloma. Of these, 24 would be " carcinomas *in situ* " and 2 papillary carcinomas if Stewart's classification were used. Adair (1953) gave a relative incidence of 53 per cent benign duct papillomas to 47 per cent malignant. MacLean (1953) regarded 1·6 per cent of breast cancers as papillary in a review of 546 breast cancers at the Winnipeg General Hospital.

In the male breast, duct papilloma is extremely rare, only 3 reported cases could be traced by Geschickter (1943), and Moroney (1946) has reported one subsequent case of multiple duct papilloma.

Carcinoma of the male breast, though also rare is much less so than duct papilloma and Geschickter found that a higher proportion of breast cancers are papillary in the male than in the female. Treves and Holleb (1955), reviewing 146 cases of male breast cancer at the Memorial Hospital, New York, from 1924 to 1954, found 12 cases of papillary carcinoma (8·2 per cent).

Only two cases of duct papilloma in the male breast with evidence of malignant change can be traced in the literature : one was reported by Geschickter (1943) and the other by Wainwright (1927). The present case falls in this group in that

---

EXPLANATION OF PLATE

FIG. 1.—Naked eye appearance of cyst with papilloma arising from wall.

FIG. 2.—Low power view of duct papilloma of breast showing at bottom left some invasion of base.

FIG. 3.—High power intimate structure of duct papilloma.

1

2

3

the histology of the tumour appears to be that of a duct papilloma even though there is some apparent spread in the base.

Clinically the long history before this patient presented for treatment is quite in keeping with previously reported cases ; Cheatle and Cutler (1931) considered the average delay in seeking advice to be 25 months, and found that 75 per cent of papillary tumours of the male have a serosanguinous discharge, 33 per cent pain.

The treatment of benign (papillary) tumour in the male breast seems to require simple mastectomy ; malignant tumours are treated just as are cancers of the female breast.

The prognosis of cancer of the breast is well known to be less favourable in the male than in the female, yet Treves and Holleb (1955) have shown that papillary carcinoma has an excellent prognosis, 6 of their 12 cases mentioned above being without recurrence 5 years after radical mastectomy ; this compares very favourably with their overall five-year survival rate of 29·1 per cent.

### SUMMARY

1. A case of duct papilloma in the male breast with early malignant change is reported.

2. The literature is reviewed regarding the incidence, pathology and prognosis of papillary tumours in the male breast.

I wish to express my gratitude to Professor C. V. Harrison and Dr. A. G. E. Pearse of the Department of Pathology, Postgraduate Medical School, London, for their opinions, advice and encouragement.

My thanks are due to Dr. A. Celestin and Dr. E. H. Madge who had charge of the patient and Dr. R. Lavoipierre, Director of Medical Services, Mauritius for permission to publish this case.

### REFERENCES

Adair, F. E.—(1953) *Surg. Clin. N Amer.*, **33**, 313.

Cheatle, G. L. and Cutler, M.—(1931) 'Tumours of the Breast'. Philadelphia (J. B. Lippincott & Co.).

Geschickter, C. F.—(1943) 'Diseases of the Breast'. Philadelphia (J. B. Lippincott & Co.).

Haagensen, C. D., Stout, A. P., Phillips, J. S.—(1951) *Ann. Surg.*, **133**, 18.

MacLean, W. A.—(1953) *J. int. Coll. Surg.*, **20**, 430.

McClure, J. A. and Higgins, C. C.—(1951) *J. Amer. med. Ass.*, **461**, 1.

Moroney, A.—(1946) *Brit. J. Surg.*, **33**, 397.

Paré, Ambroise, quoted by—Somerville, P. (1952) *Ibid.*, **39**, 296.

Snyder, W. H. and Chaffin, L.—(1955) *Arch. Surg., Chicago*, **70**, 5.

Stewart, F. W.—(1950) Armed Forces Institute of Pathology, Washington, D.C. Atlas of Tumour Pathology Section IX, Fascicle 34.

Treves, N. and Holleb, A.—(1955) 'Cancer of Male Breast'. Cancer, Philadelphia, 1239.

Wainwright, J. M.—(1927) *Arch. Surg., Chicago* **14**, 836.